# Dissecting the Mutational Landscape of Cutaneous Melanoma: An Omic Analysis Based on Patients from Greece

**DOI:** 10.3390/cancers10040096

**Published:** 2018-03-29

**Authors:** Georgia Kontogianni, Georgia Piroti, Ilias Maglogiannis, Aristotelis Chatziioannou, Olga Papadodima

**Affiliations:** 1 Metabolic Engineering and Bioinformatics Group, Institute of Biology, Medicinal Chemistry and Biotechnology, National Hellenic Research Foundation, 11635 Athens, Greece; gkontogianni@eie.gr (G.K.); gpiroti@eie.gr (G.P.); 2 Department of Digital Systems, School of Information and Communication Technologies, University of Piraeus, 18534 Piraeus, Greece; imaglo@unipi.gr; 3 e-NIOS Applications Private Company, 17671 Kallithea, Greece

**Keywords:** cutaneous melanoma, whole exome sequencing, somatic mutations, SNPs, FFPE, bioinformatics

## Abstract

Melanoma is a lethal type of skin cancer, unless it is diagnosed early. Formalin-fixed, paraffin-embedded (FFPE) tissue is a valuable source for molecular assays after diagnostic examination, but isolated nucleic acids often suffer from degradation. Here, for the first time, we examine primary melanomas from Greek patients, using whole exome sequencing, so as to derive their mutational profile. Application of a bioinformatic framework revealed a total of 10,030 somatic mutations. Regarding the genes containing putative protein-altering mutations, 73 were common in at least three patients. Sixty-five of these 73 top common genes have been previously identified in melanoma cases. Biological processes related to melanoma were affected by varied genes in each patient, suggesting differences in the components of a pathway possibly contributing to pathogenesis. We performed a multi-level analysis highlighting a short list of candidate genes with a probable causative role in melanoma.

## 1. Introduction

Cutaneous melanoma (CM) is a malignant tumour of epidermal melanocytes, the cells producing melanin located in the basal layer of the epidermis. Melanocytes originate from the neural crest, a transient embryonic structure consisting of highly migratory pluripotent cells, which will give rise to a number of cell types including melanocytes [1]. Although CM accounts for less than 5% of skin cancer incidence, it is responsible for the majority of deaths related to skin cancer due to its highly aggressive nature [2].

During the last decades, a continuous increase of CM frequency rates has been observed in Caucasian populations worldwide, making CM the most rapidly increasing cancer. CM incidence varies significantly within populations from different geographic regions, with Australia and New Zealand presenting the highest incidence rates worldwide. In Europe, the rates are lower, but still showed a three-fold to five-fold increase over the last decades [3]. CM occurrence differs a lot in European countries, with Switzerland showing the highest rate and Greece belonging to the group of low-incidence countries [4,5].

CM development is a complex, multi-factorial process involving the interplay of genetic and environmental risk factors. The most well-established environmental risk factor is the exposure to ultraviolet radiation (UVR) [2]. Regarding the genetic background, several susceptibility genes have been identified, including highly penetrative genes such as *CDKN2A*, the first familial melanoma gene identified [6,7] which is found mutated in approximately 40% of melanoma high-density families. Other less frequent mutations have been identified in genes of high or more moderate penetration, including *CDK4* and the more recently described *BAP1*, *TERT*, *POT1*, *ACD*, *TERF2IP* and *MITF* [8]. Genome-wide association studies (GWASs) have also revealed numerous recurring single nucleotide polymorphisms (SNPs) associated with melanoma risk [9,10,11,12]. Further characterisation of the genetic risk factors in different patient populations could help develop more efficient prevention strategies and improve strategies for early diagnosis.

In the last decade, important steps towards characterising the somatic mutational landscape of melanoma have been achieved [13,14]. Identifying causative melanoma mutations is of great importance in order to understand the molecular basis of melanoma genesis and progression. Towards this end, next generation sequencing (NGS) technologies are a valuable tool and have been exploited in a number of recent studies, comparing sequencing data from melanoma tissue and a matched normal control in order to identify somatic mutations [13,15,16,17,18,19]. In such approaches, discriminating driver mutations from passenger ones remains a challenge from both the experimental as well as the bioinformatics point of view [20,21,22,23,24]. Especially in the case of melanoma, which is one of the cancers with the highest mutation burden and heterogeneity, this problem is even more difficult to address, due to the confounding impact of melanoma’s high mutation rate.

The majority of studies utilising NGS for the characterisation of melanoma genome are using fresh-frozen tissue samples [25]. Nevertheless, formalin-fixed, paraffin-embedded (FFPE) tissue is the most common specimen available for molecular assays on tissue after diagnostic histopathological examination, and a number of archived samples are available for retrospective studies. The major limitation of using FFPE samples in molecular biology analyses is that nucleic acids isolated from FFPE tissue often suffer from degradation and chemical modifications. Particularly in the case of primary melanomas, which generally have a small size at the time of diagnosis, most of the tissue is used for diagnostic evaluation, rendering an additional issue of tissue-availability. However, several protocols have been developed to improve the isolation of DNA/RNA from FFPE specimens [26,27] as well as specialised library construction methods allowing NGS-based analyses starting from nucleic acids of limited quantity and poor quality [28]. Studies evaluating the quality of genomic variant calling and/or gene expression quantification by NGS, based on nucleic acids isolated from FFPE specimens as compared to fresh–frozen tissue, revealed that this approach, although challenging, can produce accurate data [28,29,30,31,32].

In this work, we aimed to exploit FFPE samples for the identification of somatic mutations and germ-line variants in patients with primary melanomas by exome sequencing. Regarding the genetic factors involved in melanoma susceptibility, a number of studies concerning melanoma patients from the Greek population have been reported [12,33]. Still, the characterisation of melanoma somatic mutations in Greek patients has been limited and, to the best of our knowledge, this is the first attempt to characterise the somatic mutational profile at the exome level of primary melanoma patients in Greece.

## 2. Results

### 2.1. Sequencing Data

In this study, we used FFPE tissue from nine patients with cutaneous melanoma after excisional biopsy. Our samples consisted of the paired tumour and surrounding normal skin that was used as control. All patients had no reported family history of melanoma and all examined melanoma tissues were from the primary lesion. 

The whole exome sequencing (WES) data were aligned to the human genome, with an average alignment rate of >91%, an average sequence coverage of >100× and over 96% of targets with at least 20× coverage, enabling the achievement of the intended mutational profile [31,34,35,36]. Only one sample attained a lower score in terms of coverage (normal sample for patient 8), but this did not affect further analysis, since high coverage is necessary in the tumour samples to overcome endogenous heterogeneity. Table 1 summarises the calculated alignment scores and sequencing depths for all the samples.

### 2.2. Identification of Germ-Line Variation

Aiming to examine whether the patients had germ-line variations on possible melanoma susceptibility loci, we focused on a panel of SNPs previously reported to be associated with CM risk. In particular, a list of SNPs from the GWAS catalog database [37] enriched by putative melanoma risk SNPs based on the MelGene database [10,33] was assessed. The analysis was restricted to 22 SNPs located in exon regions, since all SNPs on intronic or intragenomic regions were excluded because our data was derived from exome sequencing. Table 2 demonstrates the germ-line SNPs associated to melanoma risk found in the analysed patients. The relevant genes include pigmentation associated genes (*SLC45A2*, *OCA2*, *TYR*), as well as cell cycle and DNA repair genes (*ATM*, *CDKN2A*, *ERCC5*). Specific melanoma susceptibility alleles [38,39,40,41,42,43] were found in a number of patients.

### 2.3. Identification of Somatic Coding Mutations

**Figure 1:**
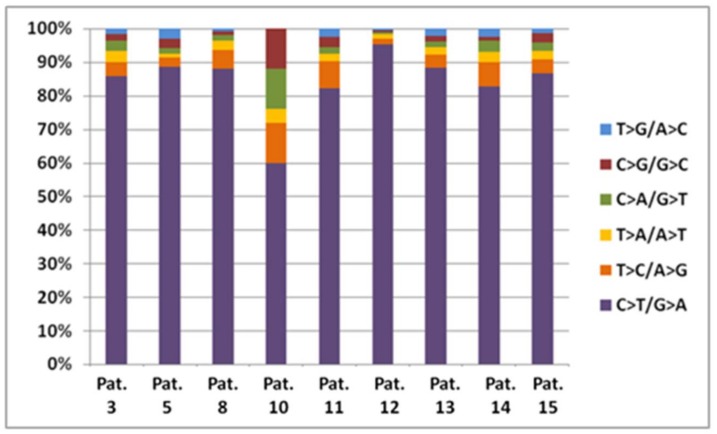
Mutation spectrum for each patient. C > T transitions account for 85.6% of the mutations (median rate).

In order to identify somatic mutations, we used the MuTect algorithm [35] which detects somatic variations at low allelic fraction with high sensitivity and low false positive rate, based on the paired analysis of tumour and matched-normal sequencing data [44]. We identified a total of 10,030 somatic mutations in all patients (see Table S1). The majority of patients had comparable numbers of somatic mutations (median: 589), with the exception of two patients: patient 12, who had a total of 5324 somatic mutations, affecting 3752 genes, and patient 10 who had 27 somatic mutations, affecting 23 genes. Table 3 displays the number of somatic mutations for all the patients, the mutation frequency per Mb, along with the number of non-synonymous mutations and the relevant number of affected genes. In particular, 3955 protein-altering somatic mutations were identified. Excluding patient 10, the median mutation frequency was 12.75 mutations/Mb (ranging from 10.1 to 105.7), which is in agreement with the previously reported mutation burden of melanoma genome, which is considered one of the highest among cancer genomes [45]. Regarding the sample from patient 10, it was the only case of acral melanoma, which has been reported to have markedly less somatic mutations. Next, we analysed the distribution of somatic substitutions per base change and all patients, except patient 10, showed a UVR characteristic mutational spectrum with a high ratio of C > T transitions (median rate 85.6%), which has been reported to characterise sun-exposed melanomas [25,46] (Figure 1). 

#### 2.3.1. Characterisation of Mutated Genes and Copy Number Variations

**Figure 2:**
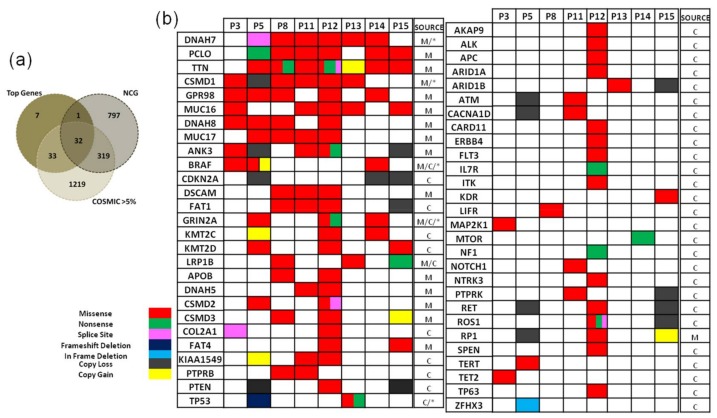
(**a**) Common genes between the 73-genes list, the 1571 Network of Cancer Genes (NCG) genes and the >5% mutated genes in melanoma from COSMIC; (**b**) Characterisation of genes carrying non-synonymous mutations based on COSMIC data. M-melanoma-associated genes (>20% mutated in COSMIC) and C-cancer-census genes (>5% in melanoma samples). * Denotes genes highlighted by MutSigCV (version 1.41).

The 10,030 mutations corresponded to 6030 unique genes, of which 2890 harboured non-synonymous mutations, most likely affecting protein functionality. Among them, 421 genes were found in at least two and 73 genes were common in at least three patients. In order to gain insights about the functional role of these common genes, the Network of Cancer Genes (NCG) [47] was accessed so as to identify all the cancer-related genes from this 73-gene top common list. NCG is a manually curated literature-based repository containing 1571 cancer genes with either known involvement in cancer or high probability of association due to statistical analysis from numerous NGS studies. Out of the 73 genes, 33 were referred to as cancer genes according to NCG, namely *DNAH7*, *PCLO*, *TTN*, *CSMD1*, *GPR98*, *MUC16*, *PKHD1L1*, *MYOM2*, *NEB*, *RELN*, *SPHKAP*, *UNC13C*, *ADCY8*, *ANK3*, *BAI3*, *CD163L1*, *CNTN5*, *COL22A1*, *DNAH14*, *EYS*, *FAT1*, *FAT3*, *FLT1*, *GRIN2A*, *KMT2D*, *PCDH18*, *PKHD1*, *SHROOM3*, *THSD7B*, *TNC*, *BRAF*, *LRP1B* and *RYR1*. In addition, the COSMIC database [48,49] was accessed to identify genes previously reported in melanoma. 65 of the 73 top common genes were previously identified in melanoma cases, with a frequency over 5%. Figure 2a demonstrates that the majority of the 73 genes, found mutated in at least three patients, either belonged to the candidate cancer gene list of NCG (containing 1571 genes) or were previously reported in COSMIC with a mutation frequency of >5% in melanoma samples (1181 genes). Among the seven genes identified only in our study, WD repeat domain 87 (*WDR87*), was found mutated in seven patients (87.5%). *WDR87* is a protein coding gene, but little is known about its function and its implication in melanoma. In COSMIC, it is found mutated in 4.2% of the samples. Additionally, we accessed TCGA’s cBioPortal database [50,51] to investigate preceding discoveries for *WDR87*. This search exposed two melanoma studies, with *WDR87* mutated in 55% and 16% of the samples examined [52,53]. Further analysis is needed to clarify the potential significance of the high mutation frequency observed for *WDR87* gene in the specific subjects.

Somatic copy number variation (CNV) was assessed using differences in sequence coverage between each tumour specimen and all same-sex adjacent skin samples utilising CNVkit, a methodology which uses both on-target and off-target reads to infer copy number consistently across the genome. This analysis revealed several CNV events in genes implicated in melanoma and reported to harbour amplifications or deletions [17,54]. Specifically, *CDKN2A* (9p21) presented a deletion signal on three of the patients, and *PTEN* (10q23) on two. In addition, *CCND1* (11q13.3) and *MITF* (3p13) were amplified in two patients and *BRAF* (7q34) was amplified in one patient (Figure 2b).

Next, we searched the whole list of 2890 genes found to contain non-synonymous mutations in at least one patient, exploring the COSMIC database which contains data of somatic mutations for specific cancer types but also data for genes causally implicated in cancer. The notable melanoma-associated mutation *BRAF V600E* was detected in three patients, while *RAS* mutations were not detected. Among the mutated genes, only BRAF, CTNNB1, NF1 and TP53 carried specific mutations that have been previously reported in COSMIC (in more than 15 cases), as shown in Table S2. We used two criteria to characterise the genes carrying non-synonymous mutations in our study: the frequency that a gene has been found mutated in melanoma and the characterisation of a gene as cancer census. Specifically, we searched for genes mutated in melanomas with a frequency >20% and in addition Cancer Census genes reported as mutated in melanoma with a frequency >5%, both based on COSMIC (Figure 2b). Furthermore, the MutSigCV algorithm (version 1.41) was used to identify significantly mutated genes, incorporating patient-specific mutation frequency with gene expression and replication time data. The small sample size prevents statistical significance in our results; still, the algorithm offers valuable information, by prioritising genes with putative significant mutations (denoted with * in Figure 2b), mainly after correcting for gene-specific mutation rates. It should be noted that among the most frequently mutated genes in our results, there are several constantly found mutated in cancer (e.g., *PCLO*, *TTN*) that are considered non-oncogenic [23]. Still, focusing only on the top melanoma census genes from COSMIC, the majority of them are also mutated in the analysed cases (Figure S1).

#### 2.3.2. Bioinformatics Pathway Analysis

**Figure 3:**
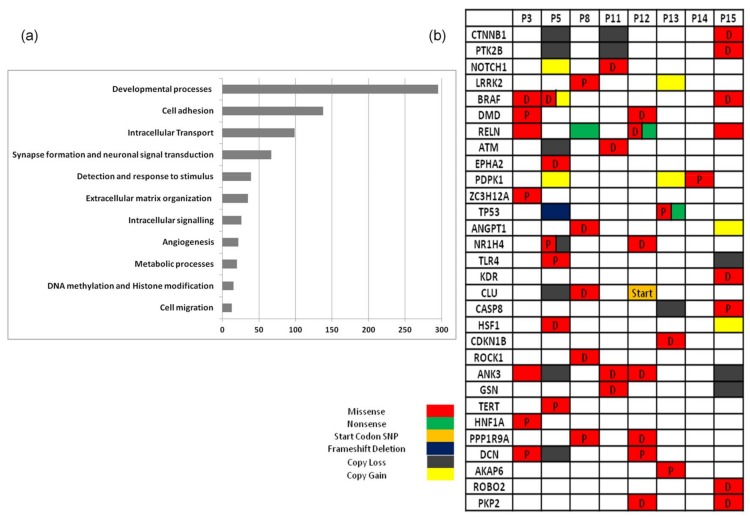
(**a**) Statistically significant biological processes with the corresponding number of genes found as mutated in at least one patient; (**b**) 30 top-prioritised mutated genes, D-probably damaging and P-possibly damaging mutation, as predicted by PolyPhen2.

In order to examine whether the genes found to carry somatic mutations were related to specific biological mechanisms, we performed enrichment analysis on the union of non-synonymous mutations for all the patients, particularly missense, nonsense, frame-shifting, splice site and non-stop mutations. Excluding the genes that were solely mutated in patient 12 (1303 genes) to avoid patient-specific bias, as well patient 10, who harboured very few mutated genes, a starting list of 1587 genes was obtained. Aiming to focus on genes putatively contributing to melanoma pathophysiology and filter out those carrying non-significant mutations, we applied two filtering steps at the 1587 gene list. Firstly, taking into account the predicted impact of each mutation on protein functionality, as predicted by PolyPhen2 tool [55], we excluded all genes carrying neutral mutations. Moreover, we explored whether these genes are expressed in melanoma through TCGA’s cBioPortal dataset, and retained for pathway analysis only those appearing to have at least low expression in over 30% of the cases. These filtering steps reduced the list to 769 genes, which were used as input for enrichment analysis based on gene ontology (GO) and Reactome. The BioInfoMiner tool was used and statistically significant enriched terms were revealed, which were grouped according to their biological relevance in Figure 3a. A great number of genes fall in the categories of developmental processes (295 genes) and cell adhesion (138 genes). Interestingly, 67 genes were related to neural system characteristic mechanisms, as indicated by GO terms such as ‘neuronal action potential’, ‘synapse organisation’, ‘regulation of myelination’ and ‘neuron projection guidance’, grouped under the label ‘synapse formation and neuronal signal transduction’. With the scope of distinguishing putatively causative genes, we focused on those with implication in diverse cross-talking biological processes, reflecting genes with a central role in cellular physiology. For this reason, we performed topological analysis using BioInfoMiner, which exploits semantic information to detect and rank genes based on their centrality, as described in different databases (e.g., GO and Reactome). This analysis resulted in a short list of genes (Figure 3b) with possible causal implications in melanoma. Interestingly, in the proposed list there are several genes with a well-established connection to cancer, like *BRAF*, *ATM* and *TP53*, but also others like *PDPK1* and *DMD* which could represent intriguing, yet poorly explored targets for further evaluation and possibly cancer treatment. Particularly, *PDPK1* (3-phosphoinositide dependent kinase 1) was found altered in three patients, two of them carrying a gene amplification and one carrying a possibly damaging point mutation. Regarding *DMD* (Duchenne muscular dystrophy), it is a long gene of 2.5 Mb, located on chromosome X. In two patients, *DMD* was found containing protein-altering point mutations. It is worth mentioning that significant pathways are enriched by different genes in each patient, suggesting that the great diversity observed in genes affected by somatic mutations could reflect deregulation of common molecular mechanisms. Indeed, regarding processes with an established role in melanoma genesis and progression, such as the MAPK pathway and cell cycle (Table S3), all patients are found to have at least one mutated gene annotated by GO to the aforementioned biological processes. The fact that all these genes are expressed and bear damaging mutations supports their potential implication in a malfunctioning mechanism contributing to melanoma.

## 3. Discussion

In this study, we report the characterisation of somatic mutations and germ-line variants in patients with primary melanomas from Greece by exome sequencing analysis. This is the first study, to our knowledge, where primary CM tissue from a low-incidence, southern European country is analysed at the exome level. In particular, FFPE tissue paired samples were used. Despite the limitations of using FFPE samples in NGS analyses, they represent a valuable source of knowledge that needs to be exploited, especially in the case of CM, where clinical practice renders fresh–frozen primary tissue availability limited. Towards this end, the present work consists of a pilot study aiming to overcome technical difficulties and establish bioinformatics workflows for the exploitation of NGS approaches on FFPE clinical samples. Our goal is to expand this analysis to a greater number of patients, aiming to study any possible associations between germ-line and somatic alterations in melanoma patients. In the present analysis, the consequences of the fixation procedure were minimised, ensuring the validity of the presented results, at the cost of the inevitable loss in sensitivity. In spite of the limited number of patients analysed, we performed a multi-level analysis, exploiting vastly established databases and state-of-the-art tools to incorporate information aiming at a better understanding of the underlying mechanisms involved with melanoma. We present a short list of candidate genes with probable causative role in CM, which contains both well-known melanoma-associated genes, but also potential new players, such as *PDPK1* and *DMD*. *PDPK1* was originally characterised as a serine-threonine kinase, phosphorylating and activating *AKT* [56]. *PDPK1* is a key element at the crossroad of signal transduction pathways such as Ras/MAPK pathway and Myc-cascade, in addition to PI3K/AKT [57]. Furthermore, *PDPK1* is frequently amplified at the gene level or over-expressed in several tumour types [58,59], including melanoma [60]. As far as *DMD* is concerned, it was recently reported as a tumour suppressor in cancers, featuring myogenic programmes [61]. In melanoma cell lines, the *DMD* gene was found with deletions while the protein was frequently absent or down-regulated [62]. In addition, a recent study based on genomic data from public repositories of diverse cancer types, showed that *DMD* expression was decreased in the majority of the analysed tumours. Specifically in the case of melanoma, *DMD* was down-regulated as compared to benign nevi that already showed a reduced expression compared to normal skin [63].

## 4. Materials & Methods

### 4.1. Melanoma Samples

All samples were acquired in the context of the 12CHN-204 PROMISE project, under the strict conformity to the rules of the call. The samples derived from FFPE tissue blocks from excisional biopsies histopathologically confirmed as melanomas. Areas from tumour and adjacent healthy skin tissue were assessed and separated by a pathologist. Paired tissue samples, tumour and normal samples respectively, from nine patients, both male and female, with cutaneous melanoma were collected.

### 4.2. DNA Extraction and Exome Sequencing

DNA was isolated from the samples using QIAamp DNA FFPE Tissue protocol from QIAGEN (Hilden, Germany), with several modifications to deparaffinisation, washing and proteinase K digestion steps, to ensure better quality and higher quantity of the extracted DNA. More specifically, deparaffinisation of FFPE samples was performed with xylene (2 times) at 56 °C for 3 min and the precipitate was sequentially washed with 100%, 70% and 50% ethanol [27]. Proteinase K digestion was performed at 56 °C while stirring the samples and the incubation time was increased to 3 days with daily re-addition of proteinase K. The quantity and purity of the samples were checked using NanoPhotometer (IMPLEN, Munich, Germany). The extracted DNA was prepared and captured with the Agilent SureSelect Human All Exon 50 Mb kit (Agilent SureSelect v5, Santa Clara, CA, USA) and whole exome sequencing was performed on an Illumina HiSeq 4000 sequencer (San Diego, CA, USA), as paired-end reads.

### 4.3. Raw Data Analysis

Bioinformatic data analysis was performed utilising various state-of-the-art tools, as previously presented [64]. For this study, we used version 3.6 of GATK [65], which incorporates somatic SNP calling with somatic indel (insertions & deletions) calling, as carried out by MuTect2 [35] and Indelocator [66], comparing the tumour-normal pairs in order to characterise somatic mutations. Strand-specific artefacts, possibly due to DNA damage resulting from formalin fixation and storage time, were excluded from further analyses. Germ-line variants were identified using the HaplotypeCaller tool, by comparing the normal samples with the reference sequence, and specific coding SNPs were investigated from a known panel of germ-line variants associated with melanoma based on GWAS studies and established databases [10,33,37], focusing on those found on coding regions. Annotation of all mutations was performed using Oncotator [67]. Significantly mutated genes among the patients were identified using MutSigCV (version 1.41) [23], which ranks the genes by estimating a background mutation rate (BMR) through the number of silent versus non-coding mutations in the gene and the surrounding regions. BioInfoMiner [68] was used for the functional analysis of the mutated genes, so as to identify the molecular pathways influenced by these mutations, and to isolate the genes with central role, implicated in diverse and major mechanisms from various vocabularies. For the functional prediction of the somatic mutations we utilised dbNSFP [69] through Oncotator, a database that provides information for functional predictions and annotations for human non-synonymous variants. Copy number variation (CNV) analysis was performed using CNVkit [70], which specialises on CNV detection on targeted DNA sequencing. Expression of genes was evaluated through TCGA’s cBioPortal datasets. Genes with TPM (transcripts per million) between 0.5 and 10 were considered to have low expression, between 11 and 1000 medium, and if TPM was over 1000, the genes were considered as highly expressed in a given case. Overall, genes were considered as expressed when encompassing at least low expression in over 30% of the cases.

## 5. Conclusions

In this study, we present the characterisation of somatic mutations by WES analysis of paired tissue from FFPE melanoma samples. A number of the identified mutated genes were previously found in melanoma, including established, cancer-related genes. Our multi-level analysis highlights a short list of candidate genes with a probable causative role in melanoma that could be promising targets for future investigation.

## Figures and Tables

**Table 1:** Sequencing characteristics for all the samples.

Patients	3	5	8	10	11	12	13	14	15
**Normal**									
Alignment rate (%)	96	84.2	65.6	93.8	96.8	96.8	97.4	87.9	95.6
Average sequencing depth on target	129.8	93.9	72.2	103.9	101.5	123.3	117.4	102.5	128.4
Fraction of target covered by >20× (%)	97.37	96.37	94.41	96.44	95.03	97.53	97	97.38	97.96
**Tumour**									
Alignment rate (%)	95.4	89.5	93.6	92.6	96.7	96.2	96.8	88.8	92
Average sequencing depth on target	118.9	100.8	102.2	111.5	111.4	104.8	111.9	102.2	120.7
Fraction of target covered by >20× (%)	97.4	95.86	96.48	96.75	96.74	96.62	96.16	96.3	96.66
Average sequencing depth on target		108.7							

**Table 2:** Germ-line single nucleotide polymorphisms (SNPs) putatively associated with melanoma, based on genome-wide association studies (GWAS) and MelGene databases.

dbSNP_ID	Gene	Chr	Variant Classification	Ref. Allele	MA Allele	# Hom. Ref.	# Hom. MA	# Heter.
**rs1801516**	ATM	11	Missense Mutation	G	A	6	0	3
**rs11515**	CDKN2A	9	3′UTR	C	G	0	7	2
**rs16891982**	SLC45A2	5	Missense Mutation	C	G	0	9	0
**rs17655**	ERCC5	13	Missense Mutation	G	C	7	0	2
**rs1800407**	OCA2	15	Missense Mutation	G	A	8	0	1
**rs1042602**	TYR	11	Missense Mutation	C	A	2	2	5

The corresponding gene, chromosome position, classification type, reference allele (Ref.), Melanoma-associated (MA) allele and the number of patients in Homozygous/Heterozygous state (Hom./Heter.), are shown in the relevant columns.

**Table 3:** Somatic mutation characteristics for each patient.

Patients	3	5	8	10	11	12	13	14	15
Number of Mutations	522	693	901	27	935	5324	511	589	528
Frequency per Mb	10.4	13.8	17.9	0.5	18.6	105.7	10.1	11.7	10.5
Non-synonymous	226	284	387	18	364	2036	222	224	193
Genes with non-synonymous	216	274	360	16	338	1634	201	218	185

## Supplementary Materials

The following are available online at http://www.mdpi.com/2072-6694/10/4/96/s1, Table S1: Total somatic mutations for all the samples; Table S2: Recurrent mutations based on COSMIC; Table S3: Biological processes from GO (MAPK pathway and Cell cycle), with the corresponding mutated genes in each patient; Figure S1: Top Census genes in melanoma from COSMIC database and the type of mutation found in all patients.

## Abbreviations

BMR	background mutation rate
CM	cutaneous melanoma
CNV	copy number variation
COSMIC	Catalogue of Somatic Mutations in Cancer
FFPE	formalin fixed paraffin embedded
GWAS	genome wide association study
Indel	insertion/deletion
NCG	Network of Cancer Genes
NGS	next generation sequencing
SNP	single nucleotide polymorphism
TCGA	The Cancer Genome Atlas
TPM	Transcripts per million
UTR	un-translated region
UVR	ultraviolet radiation
WES	whole exome sequencing
Nucleotides	A-adenine, C-cytosine, G-guanine, T-thymine
Amino-Acids	E-glutamic acid, V-valine
