# Supplementary material for: Dissecting the Mutational Landscape of Cutaneous Melanoma: An Omic Analysis Based on Patients from Greece
Source: Cancers (Basel). 2018 Mar 29;10(4):96. doi: 10.3390/cancers10040096 (PMC5923351; doi:10.3390/cancers10040096)
Supplement: Supplementary file 1 [file cancers-10-00096-s001.zip › Figure S1.pdf]

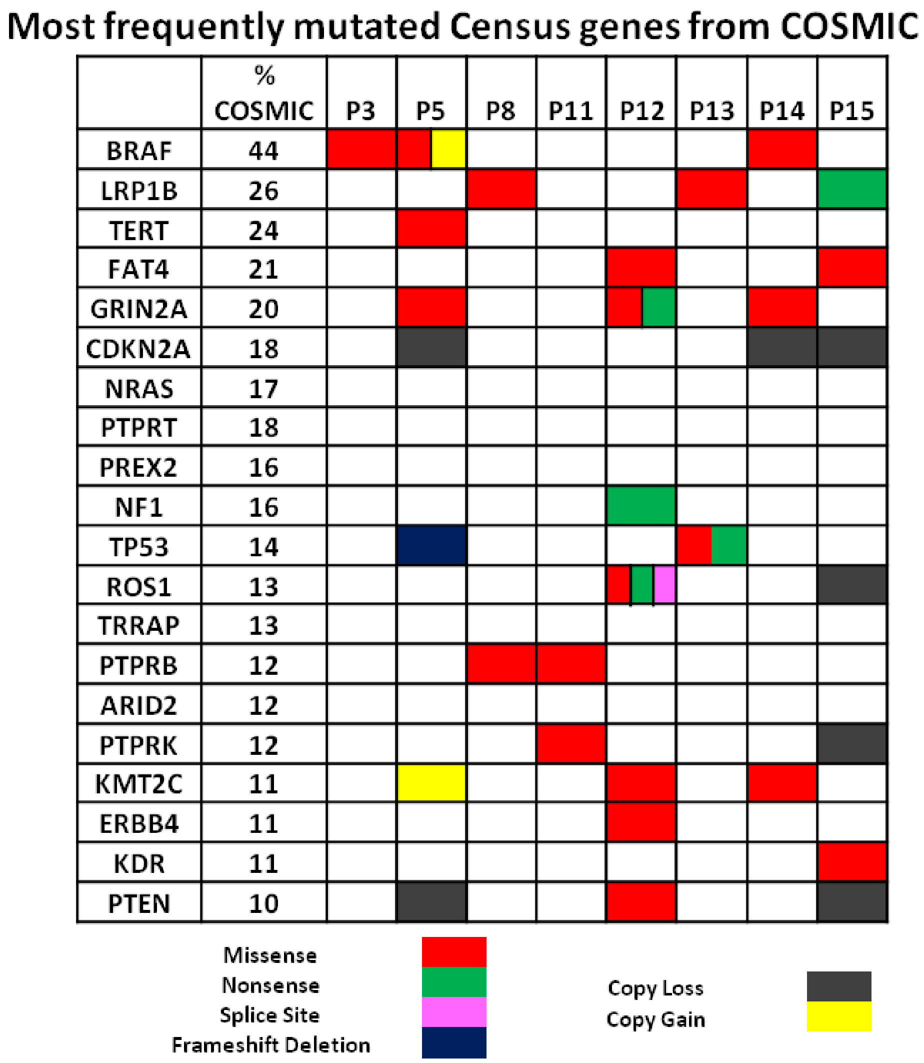

Figure S1: Top Census genes in melanoma from COSMIC database and the type of mutation found in all patients
